# Case Report: Rapidly progressive bilateral pleural effusions in a 12-year-old girl with multisystem inflammatory syndrome who was successfully treated with prednisolone and cyclosporine

**DOI:** 10.3389/fped.2025.1633264

**Published:** 2025-10-24

**Authors:** Takeshi Yamamoto, Kentaro Okunushi, Kei Watanabe, Yutaka Hirata, Hironori Sato, Koo Nagasawa, Taiji Nakano, Tomozumi Takatani, Hiromichi Hamada

**Affiliations:** Department of Pediatrics, Graduate School of Medicine, Chiba University, Chiba, Japan

**Keywords:** multisystem inflammatory syndrome in children (MIS-C), Hemophagocytic lymphohistiocytosis (HLH), serositis, cyclosporine, COVID-19

## Abstract

This case report explores a unique presentation of macrophage activating syndrome (MAS) as well as multisystem inflammatory syndrome in children (MIS-C), a complication of COVID-19, that was characterized by polyserositis with massive pleural effusions and edema. A 12-year-old girl had cervical pyogenic lymphadenitis, dyspnea, and cough in addition to the bilateral conjunctivitis, facial edema, erythema on both cheeks, and edema in the extremities. Initial laboratory investigations revealed a white blood cell count of 5.8 × 10^9^/L, hemoglobin 9.7 g/dl, platelet count 75 × 10^9^/L, C-reactive protein level 149 mg/L, serum aspartate aminotransferase 73 U/L, alanine aminotransferase 64 U/L, fibrinogen 446 mg/dl. The dyspnea and cough rapidly worsened and a chest x-ray demonstrated massive plural effusions bilaterally. An echocardiographic study showed slight pericardial effusion and a normal cardiac ejection fraction. Her condition, fever, high serum triglyceride, high serum ferritin level, hemophagocytosis, low NK cell activity, and high serum soluble IL-2R level were attributed to MAS. We started intravenous administration of prednisolone (2 mg/kg). The respiratory distress and pleural effusions showed little change 2 days after starting prednisolone, so we added oral cyclosporine (5 mg/kg/day) based on the HLH-2004 protocol. Soon after starting cyclosporine, the respiratory distress and oxygenation improved and the pleural effusions significantly decreased. One month before admission, the patient's mother had fever and respiratory distress due to PCR-confirmed SARS-CoV-2 infection during the omicron variant wave in Japan. At that time, the patient also had fever. SARS-CoV-2 titers were subsequently tested, revealing that both anti-N antibodies and anti-S protein antibodies were positive. In 2021, there are few patients with COVID-19 in Japan, so the antibody titers were an important diagnostic tool in this period. Taking together these findings, we diagnosed her condition as MIS-C. The case highlights the complex overlap between MIS-C and MAS, with immunosuppressive therapy, particularly cyclosporine, playing a critical role in the management of severe cases.

## Introduction

Multisystem inflammatory syndrome in children (MIS-C) is a complication of COVID-19 that is characterized by multi-organ dysfunction and presents with symptoms similar to Kawasaki disease (KD) (1, 2). In MIS-C, circulatory dysfunction and gastrointestinal disorders are more severe, whereas coronary artery aneurysms are less common. Not all cases with MIS-C are characterized by multi-organ dysfunction, there is a phenotypic spectrum, ranging from febrile syndrome, KD-like illness to shock syndrome with multiple organ dysfunction and macrophage activation syndrome (MAS) (3, 4).

Hemophagocytic lymphohistiocytosis (HLH) is a syndrome of pathologic immune activation, often associated with genetic defects affecting lymphocyte cytotoxicity, called primary HLH (5). Secondary HLH is commonly triggered by infection, such as Epstein–Barr virus infection, and non-infection diseases, including malignancy and collagen diseases. Secondary HLH triggered by immune disorders is called MAS. SARS-CoV-2-associated MAS can sometimes become life-threatening. However, little information is currently available regarding MAS complicating MIS-C (6). Moreover, differences between SARS-CoV-2 associated-MAS and those associated with other causes are not known.

Here we report a case with MIS-C in which polyserositis was the predominant clinical manifestation, with dyspnea and edema as the main clinical signs. This case also met the criteria for MAS, and we believe this case provides important insights into the pathogenesis and treatment of MIS-C (7).

## Case presentation

**Figure 1:**
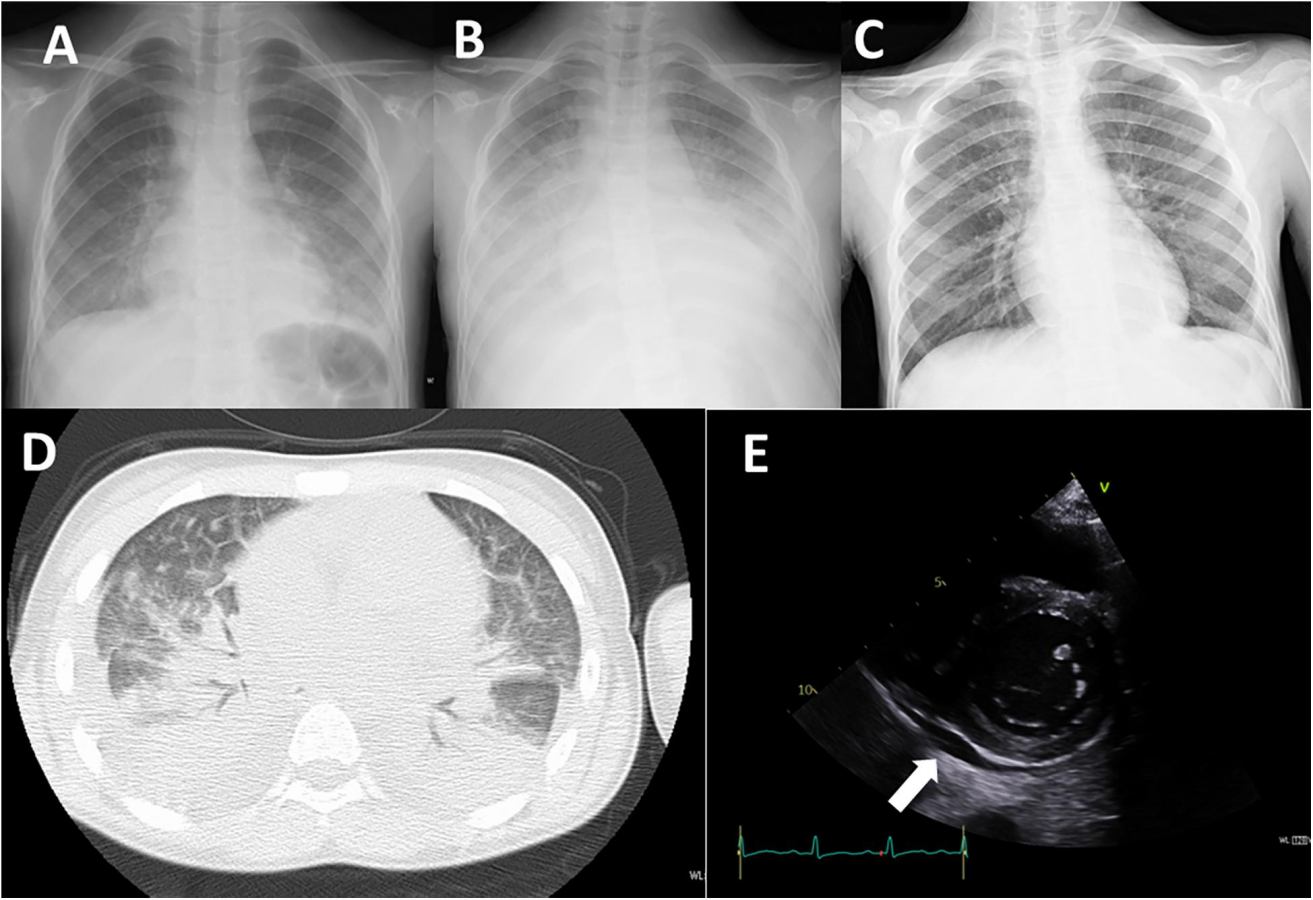
Imaging studies. **(A)** Chest x-ray on admission to the previous hospital. **(B)** Chest x-ray on admission to our hospital (day 7 of illness). **(C)** Chest x-ray on day 12 of treatment (day 19 of illness). **(D)** Chest CT image on admission to our hospital (day 7 of illness). E: Echocardiography on admission to our hospital (day 7 of illness). Arrow indicates pericardial effusion.

A 12-year-old girl had been aware of bilateral neck pain for 3 weeks prior to her first visit to the hospital. She had a fever over 38.0°C for 1 week before the visit. She was diagnosed as having cervical pyogenic lymphadenitis and was referred and admitted to the previous hospital. After starting intravenous antibiotics, she developed dyspnea and cough in addition to bilateral conjunctivitis, facial edema, and erythema on both cheeks. Her extremities had also appeared edematous. The dyspnea and cough rapidly worsened and a chest x-ray demonstrated massive plural effusions bilaterally (Figure 1).

One month before admission, the patient's mother had fever and respiratory distress due to PCR-confirmed SARS-CoV-2 infection during the omicron variant wave in Japan. At that time, the patient also had fever but was not tested for COVID-19. She received an mRNA COVID-19 vaccine 10 days before hospitalization. She had a history of mild atopic dermatitis and seasonal allergic rhinitis, but did not regularly take any medications.

On admission, her oxygen saturation was under 90% on room air. She had a respiratory rate of over 40 breath/min, heart rate 120 bpm, body temperature 37.5°C, blood pressure 99/52 mmHg, and body weight 32.6 kg (compared with 28.0 kg 1 month earlier). Oxygen saturation increased to 98% under oxygenation. On physical examination, she had an erythematous rash on both cheeks with no rash on the nose, inconsistent with butterfly rash observed in systemic lupus erythematosus. The abdomen was diffusely tender to palpation. Neither splenomegaly nor hepatomegaly were observed. Initial laboratory investigations revealed a white blood cell count of 5.8 × 10^9^/L (normal 3.8–10.1 × 10^9^/L), hemoglobin 9.7 g/dl (normal 11.9–14.9 g/dl), platelet count 75 × 10^9^/L (normal 180–440 × 10^9^/L), C-reactive protein level 149 mg/L (normal ≤3 mg/L), ferritin 2,439 ng/mL (normal 4.0–64.2 ng/mL), fibrinogen 446 mg/dl (normal 181–378 mg/dl), serum albumin 1.9 g/dl (normal 3.8–4.7 g/dl), serum aspartate aminotransferase 73 U/L (normal 15–30 U/L), alanine aminotransferase 64 U/L (normal 9–28 U/L), serum creatinine 0.36 mg/dl (normal 0.39–0.69 mg/dl), serum triglyceride 222 mg/dl (normal 30–149 mg/dl), IgG 720 mg/dl (normal 790–1,800 mg/dl), C3 126 mg/dl (normal 73–138 mg/dl), C4 7 mg/dl (normal 11–31 mg/dl), CH50 35.7 mg/dl (normal 30–40 mg/dl), sIL-2R 4,960 U/mL (normal 121–613 U/mL), IL-6 265 pg/mL (normal ≤3 pg/mL, IL-18 1,784 pg/mL (normal 36–258  pg/mL), and brain natriuretic peptide (BNP) 685 pg/mL (normal ≤18.4 pg/mL). NK cell activity was 9%, and anti-nuclear antibody was negative. A chest x-ray showed decreased transparency in both lung fields (Figure 1B). An echocardiographic study showed slight pericardial effusion and a normal cardiac ejection fraction (Figure 1E). A chest CT demonstrated massive plural effusions bilaterally (Figure 1D). Bone marrow aspiration performed on the third day of hospitalization revealed mild macrophage activation and hemophagocytosis. No significant bacterial growth was detected in standard microbiological cultures, including blood, sputum, and stool cultures. Serological testing for viral infections, potentially those associated with secondary HLH, such as Epstein–Barr virus, cytomegalovirus, and parvovirus B19, revealed a non-infectious profile, characterized by negative IgM antibodies.

**Figure 2:**
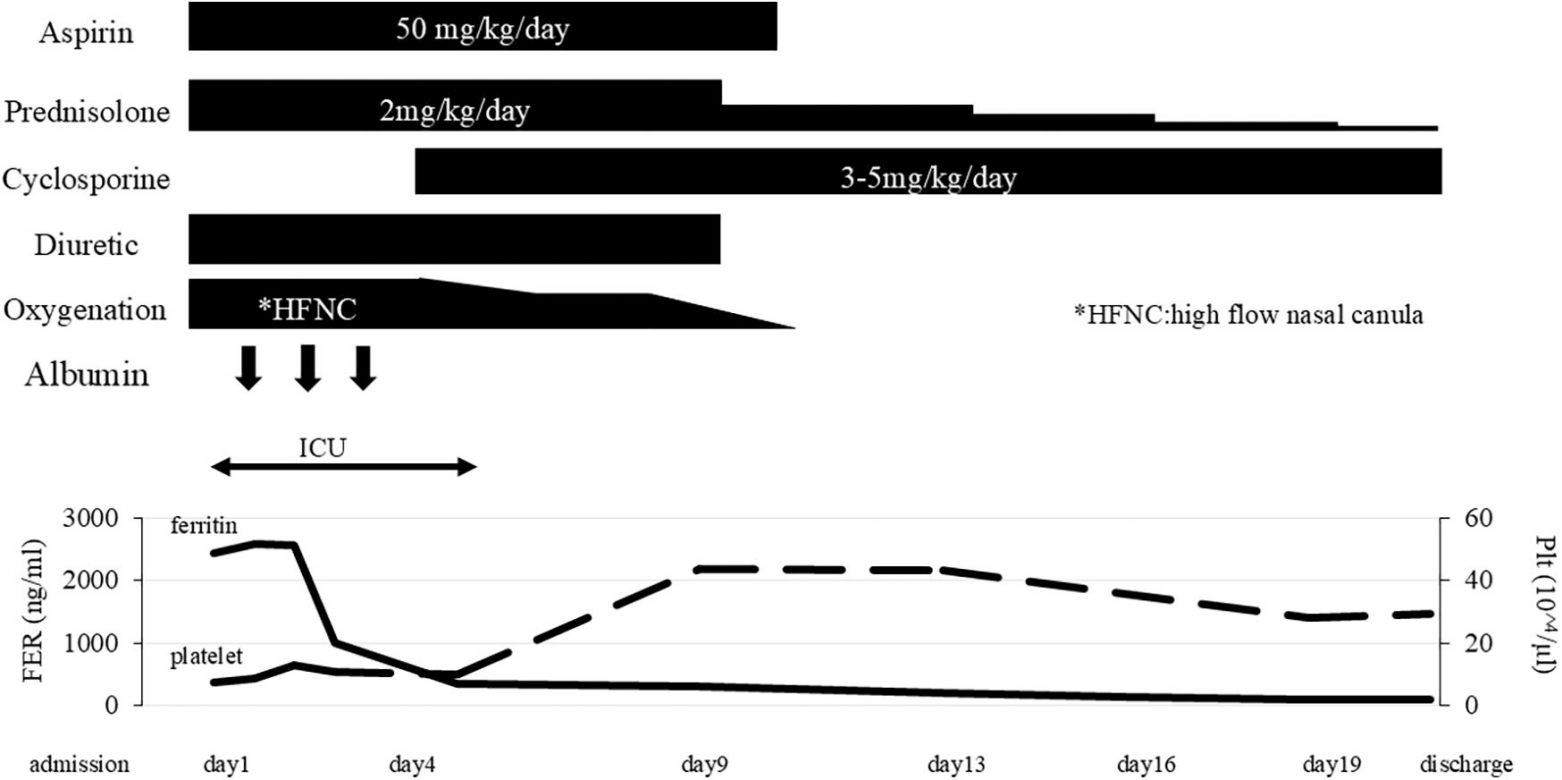
Clinical course. FER, serum ferritin level; HFNC, high-flow nasal canula: Plt, platelet count.

Since she was transferred to our hospital on a weekend, and her condition, fever, high serum triglyceride, high serum ferritin level was attributed to MAS and we immediately started intravenous administration of prednisolone (2 mg/kg). She fulfilled only three of the six principal clinical manifestations of KD. Given that differential diagnoses such as systemic lupus erythematosus and infectious diseases had not fully excluded, prednisone was selected over high-dose intravenous methylprednisolone. Aspirin (50 mg/kg/day) was given for 10 days. She responded to corticosteroid therapy, with rapid resolution of fever and a tendency toward improvement in elevated liver enzymes. However, the recovery of platelet counts was limited, in addition, the respiratory distress and pleural effusions showed little change 2 days after starting prednisolone, and hemophagocytosis, low NK cell activity, and high serum soluble IL-2R level were identified on weekday, so we added oral cyclosporine (5 mg/kg/day) based on the HLH-2004 protocol (4). Soon after starting cyclosporine, the respiratory distress and oxygenation improved along with a significant reduction of the pleural effusions and pericardial effusion (Figure 2), therefore, the chest drainage and serological analysis and bacterial or viral cultures of the pleural fluid were not performed. Furthermore, neither coronary artery aneurysm formation nor cardiac dysfunction was observed. SARS-CoV-2 titers were subsequently tested, revealing that both anti-N antibodies (2.6 AU/ml) and anti-S protein antibodies (372 U/ml) were positive. In 2021, there are few patients with COVID-19 in Japan, so the antibody titers were an important diagnostic tool in this period. Taking together her clinical findings and the evidence of SARS-CoV-2 infection, we diagnosed her condition as MIS-C.

She did not require additional treatment such as anti-cytokine therapy or salvage therapy. The pericardial effusion disappeared at admission day 9. The dose of prednisolone was gradually tapered over 3 weeks, and she was discharged with no symptoms. Cyclosporine was discontinued 1 week after discharge (Figure 2). She was discharged 21 days after starting treatment without any adverse events. There were no signs of relapse after discontinuation of the treatment during 3 years of follow-up. Moreover, no clinical or laboratory features suggestive of autoimmune diseases or immunodeficiency were observed.

## Discussion

In 2023, the Centers for Disease Control and Prevention (CDC) revised the diagnostic criteria for MIS-C (Table 1) (1). The patient met the following criteria: fever, dyspnea requiring hospitalization, ocular conjunctival hyperemia, skin manifestations, elevated CRP >30 mg/L, and low platelet counts. Recently, a detailed analysis of patients with MIS-C showed that both sIL-2R and ferritin were elevated, consistent with the findings in our patient (8). A unique feature of the clinical course in this case was that there was little evidence of cardiac failure or shock, with no clinically significant shock, circulatory failure, or decreased left ventricle ejection fraction observed by echocardiography, though elevated BNP could be considered a subclinical finding (1–4). Also, she had no serious diarrhea or vomiting. We hesitated to diagnose MIS-C on the first day of admission, but MIS-C is an acute systemic vasculitis, and this patient's clinical symptoms such as pleural effusion and edema reflected the condition (9).

**Table 1:** Diagnostic criteria comparison: MIS-C (wHO and CDC) and HLH-2004.

Category	MIS-C (WHO)	MIS-C (CDC)	HLH (HLH-2004)	This Case
Fever	≥3 days	≥24 h or report of subjective fever	≥7 days (persistent high fever)	9 days
Age	0–19 years	<21 years	Any age	12 years
Multisystem involvement	≥2 systems (cardiac, renal, respiratory, hematologic, gastrointestinal, dermatologic, or neurologic)	≥2 systems (cardiac, renal, respiratory, hematologic, gastrointestinal, dermatologic, or neurologic)	Not part of formal criteria, but often liver, CNS, spleen	respiratory, dermatologic
Cardiac involvement	May include myocarditis, shock, coronary involvement	May include myocarditis, shock, coronary involvement	May include myocarditis, shock, coronary involvement	No involvement
Cytopenia	May occur	May occur	Cytopenias in ≥2 lineages: Hb <9, platelets <100, neutrophils <1.0 × 10⁹/L	platelets 75 × 10⁹/L
Hemophagocytosis	No required	No required	Demonstrated in bone marrow, spleen, or lymph nodes (supportive but not mandatory)	Demonstrated in boe marrow
Inflammatory markers	Elevated CRP, ESR, procalcitonin, or ferritin	Elevated CRP, ESR, fibrinogen, procalcitonin, D-dimer, ferritin, LDH, or IL-6	Ferritin ≥500 ng/mL, elevated sIL-2R	Ferritin 2,439 ng/mL, sIL-2R 4,960 U/mL
SARS-CoV-2 exposure	Confirmed or likely exposure within 4 weeks	Positive SARS-CoV-2 PCR, serology, antigen, or known exposure	Not required	SARS-CoV2-N-IgG 2.6 AU/mL
Microbiology	No other obvious microbial cause	No alternative plausible diagnoses	Exclusion of other causes recommended	No other obivious microbial cause
Other specific criteria			≥5/8 of the following: 1. Fever 2. Splenomegaly 3. Cytopenias (≥2 lineages) 4. Hypertriglyceridemia, hypofibrinogenemia 5. Hemophagocytosis 6. Low/absent NK cell activity 7. Hyperferritinemia 8. Elevated sIL-2R (CD25)	HLH-2004 criteria 6/8 1. Fever 4. Triglyceride 222 mg/dl 5. Hemophagocytosis in BM 6. NK cell activity 9% (Low) 7. Ferritin 2,349 ng/mL 8. sIL-2R 4,960 U/mL

Data for this case are listed in the rightmost column.

BM, bone marrow; CDC, Centers for Disease Control; CNS, central nervous system; CRP, C-reactive protein; ESR, erythrocyte sedimentation rate; Hb, hemoglobin; IGg, immunoglobulin G; IL, interleukin; LDH, lactate dehydrogenase; PCR, polymerase chain reaction; sIL-2R, soluble interleukin-2 receptor; WHO, World Health Organization.

There are few reports of MIS-C in which pleural effusion is the primary symptom. However, a retrospective study of 47 cases that underwent pulmonary ultrasonography reported that 93% had findings of pneumonia and 84% had findings of pleural effusion. In MIS-C with respiratory symptoms, the frequency of pleural effusion may be high when including potential cases (10).

The patient had received the SARS-CoV-2 vaccine 1 week before admission. MIS-C has not been reported as an adverse event following vaccination (11). Myocarditis following vaccination has been noted as an adverse event (12, 13). The myocarditis predominantly occurs in adolescent males after the second dose of the mRNA SARS-CoV-2 vaccine with the onset of chest pain ranging from one to five days after vaccine administration. Focusing on the age 12–17 years of age group which is the group most affected with vaccine-related myocarditis, for every million second-dose mRNA SARS-CoV-2 vaccinations, there is a risk of 8–10 myocarditis cases in females and 56–69 myocarditis in males. For this same age and same number of vaccinations administered, the predicted benefit is prevention of 14,200 COVID-19 cases, 398 hospitalizations, 109 ICU admissions, and three deaths in females and males combined (12). The risk-benefit analysis is overwhelmingly favorable for SARS-CoV-2 vaccinations for those aged 12 and above (12, 13). Additionally, a cohort study following the initiation of vaccination in children have shown a decrease in the incidence of MIS-C (14).

This case satisfied 6 of the 8 diagnostic criteria in HLH-2004 (Table 1) (5). At the time of hospitalization in this case, the condition of MAS was evident, and the initial diagnosis was MAS. The fact that some cases with MIS-C meet the criteria for MAS has been pointed out in several previous reports, and the fact that few cases with KD meet the diagnostic criteria for MAS has been noted as a difference between these two diseases (15). The largest MIS-C Latin American cohort revealed MAS in patients with MIS-C was associated with increased morbidity and mortality (6). Seventeen % of patients with MIS-C fulfilled MAS criteria. Importantly, the mortality rate in MIS-C with MAS was 12%, which was higher than those without it. Gastrointestinal and neurologic manifestations were more frequent in cases where MIS-C was complicated by MAS.

We followed the HLH-2004 recommendations of prednisolone and cyclosporine, and particularly cyclosporine given from day 3 of treatment appeared to be remarkably effective in improving respiratory status. The recommended protocol for secondary HLH on the setting of rheumatic diseases (which represents MAS) is diverse with methylprednisolone pulses, IVIG and cyclosporine for cases non-responsive to intravenous steroids. Based on the assessment of this patient's general condition, we first administered intravenous steroids and then chose cyclosporine. Immunosuppressive treatment including steroid and cyclosporin dramatically reduced the size of the pleural effusions and suppressed NK cell activity that would contribute to the exacerbation of MAS (16). Both the HLH protocol and recent evidence suggest that cyclosporine could be another initial treatment option for MIS-C (17, 18). Whether intravenous immunoglobulin (IVIg) is truly necessary in cases with MIS-C that do not fulfill the diagnostic criteria for KD remains unclear, as there have been few reports specifically describing clinical outcomes in patients with MIS-C treated without IVIg (19).

A recent proteomics analysis revealed an MAS-like phenotype in a subset of patients with MIS-C (7). Thrombocytopenic patients had increased ferritin levels, reduced leukocyte subsets, and increased soluble IL-2R in comparison with non-thrombocytopenic patients. T-cell activation and tumor necrosis factor-alpha and interferon-gamma signaling markers were inversely correlated with thrombocyte levels, consistent with a need for T cell-targeted immunosuppressants, such as cyclosporine. Since we did not measure any cytokines other than IL-18 and IL-6, we cannot further elucidate the immunological mechanisms or specific immune cell populations involved in the MAS-like inflammatory response in this case.

In conclusion, we note two key clinical implications: first, MIS-C should be included in the differential diagnosis of MAS in pediatric patients presenting with hyperinflammatory features; and second, early initiation of immunomodulatory therapy—such as prednisolone and cyclosporine—may contribute to favorable clinical outcomes. This unique case report also highlights massive pleural effusions and edema as the main manifestation of polyserositis, and presents clinical data showing that MAS might be found in severe cases with MIS-C. The standard treatment for MAS in the HLH-2004 protocol might offer a strategy for successful clinical outcomes in patients with MIS-C who have MAS.

## Data Availability

The original contributions presented in the study are included in the article/Supplementary Material, further inquiries can be directed to the corresponding author.
